# Assessing Proteinase K Resistance of Fish Prion Proteins in a Scrapie-Infected Mouse Neuroblastoma Cell Line

**DOI:** 10.3390/v6114398

**Published:** 2014-11-13

**Authors:** Evgenia Salta, Eirini Kanata, Christos A. Ouzounis, Sabine Gilch, Hermann Schätzl, Theodoros Sklaviadis

**Affiliations:** 1 Laboratory for the Research of Neurodegenerative Diseases, Center for Human Genetics, KU Leuven, O&N 4 Herestraat 49, PO Box 602, 3000 Leuven, Belgium; E-Mail: Evgenia.Salta@cme.vib-kuleuven.be; 2 Department of Pharmacy, Aristotle University of Thessaloniki, Thessaloniki GR-54124, Greece; E-Mail: ekanata@bio.auth.gr; 3 Biological Computation & Process Laboratory (BCPL), Chemical Process Research Institute (CPERI), Centre for Research & Technology (CERTH), PO Box 361, GR-57001 Thessaloniki, Greece; E-Mail: ouzounis@certh.gr; 4 Faculty of Veterinary Medicine, University of Calgary, 2500 University Dr. NW, Calgary, Alberta T2N 1N4, Canada; E-Mails: sgilch@ucalgary.ca (S.G.); hschaetz@ucalgary.ca (H.S.)

**Keywords:** prion, fish, cross-species transmission, cell culture, ScN2a

## Abstract

The key event in prion pathogenesis is the structural conversion of the normal cellular protein, PrP^C^, into an aberrant and partially proteinase K resistant isoform, PrP^Sc^. Since the minimum requirement for a prion disease phenotype is the expression of endogenous PrP in the host, species carrying orthologue prion genes, such as fish, could in theory support prion pathogenesis. Our previous work has demonstrated the development of abnormal protein deposition in sea bream brain, following oral challenge of the fish with natural prion infectious material. In this study, we used a prion-infected mouse neuroblastoma cell line for the expression of three different mature fish PrP proteins and the evaluation of the resistance of the exogenously expressed proteins to proteinase K treatment (PK), as an indicator of a possible prion conversion. No evidence of resistance to PK was detected for any of the studied recombinant proteins. Although not indicative of an absolute inability of the fish PrPs to structurally convert to pathogenic isoforms, the absence of PK-resistance may be due to supramolecular and conformational differences between the mammalian and piscine PrPs.

## 1. Introduction

Transmissible Spongiform Encephalopathies (TSEs), commonly known as prion diseases, are fatal neurodegenerative diseases affecting a wide range of species, varying from humans to rodents. The most notorious representatives include bovine spongiform encephalopathy (BSE) in cattle, scrapie in sheep and goats and Creutzfeldt Jakob disease (CJD) in humans. Even though prion research represents a highly active field, several issues concerning TSE pathogenesis and transmissibility still remain unanswered. The cellular prion protein PrP^C^, exhibits remarkable prominence in the vertebrate CNS, and has been implicated as the molecular prerequisite [1], whose structural conversion into PrP^Sc^, the abnormal isoform, can trigger a cascade of events leading to the prion pathognomonic phenotype [2].

Inter-species transmission of TSEs is limited by the so called “species barrier”. The magnitude of such a barrier reflects the ease by which prions can be transmitted from one species to another, the number of the affected animals, the duration of the incubation period, and the clinical and histopathological manifestations of the disease in the new host [3]. The structural homology, in addition to the amino acid sequence identity, between the donor and host PrP molecules, has been considered as one of the critical parameters affecting the existence and characteristics of a transmission barrier between two species [4,5,6].

**Figure 1:**
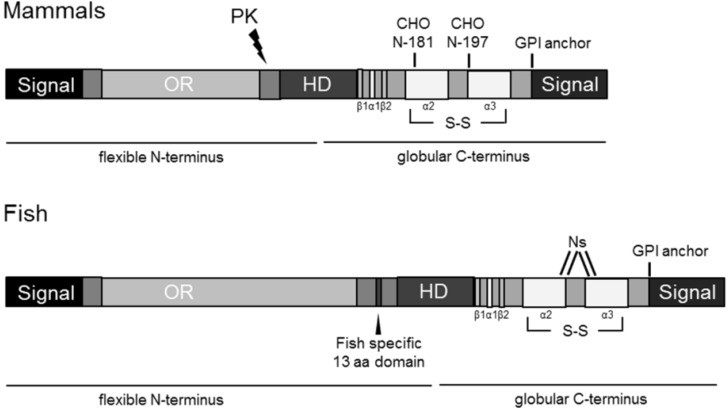
Conserved structural domains in piscine and mammalian prion proteins. Schematic illustration of prion proteins in mammals and fish. Signal, signal peptides; OR, octarepeat region; HD, hydrophobic domain; CHO *N*-, *N*-glycosylation sites; –S–S–, disulphide bond; β1, β2, β-sheets; α1, α2, α3, α-helices; GPI anchor, glycosylphosphatidylinositol anchor; PK, proteinase K cleavage site.

The identification of orthologue PrP genes in several fish species, including salmon (*Salmo salar*) [7], trout (*Onchorhynchus mykiss*) [8], zebrafish (*Danio rerio*) [9] and sea bream (*Sparus aurata*) [10] has rendered them putative candidates for hosting a prion infection. Typically, fish harbor two PrP homologues, namely PrP-1 and PrP-2, due to a teleost-specific whole-genome duplication. Although mammalian and fish prion proteins do not display great similarities at the sequence level, they do share the majority of the predicted structural motifs (Figure 1). More specifically, they all have an *N*-terminal signal peptide, a Gly-rich repeat region (that is considerably longer in fish), a central hydrophobic domain, *N*-glycosylation sites, two cysteine residues forming a disulphide bond and a glycosylphosphatidylinositol (GPI) anchor signal peptide at the *C*-terminus [10,11]. Unique to fish PrPs is a 13-residue stretch preceding the central hydrophobic region [9]. Structural homology between mammalian and piscine prion proteins could reflect a degree of functional conservation, thus, serving as a valuable tool for solving the enigma of prion protein function [12]. Indeed, a study using zebrafish, provided the first evidence of a PrP loss-of-function phenotype, revealing an indispensable role of prion proteins in cell adhesion during embryogenesis and early neuronal development [13]. More recently, data demonstrating a role for zebrafish PrP-2 in the regulation of neuronal excitability, further confirmed the intriguing functional conservation between piscine and mammalian prion proteins [14].

Current knowledge concerning prion transmission to fish is limited [15,16,17]. Previously, we reported *in vivo* observations on BSE and scrapie transmission to sea bream, an aquacultured species of significant commercial value [18]. Interestingly, sea bream force-fed BSE brain homogenate showed evidence of extensive abnormal protein deposition in fish brain. The plaques observed in this inoculation group were PrP-immunoreactive, PAS and Congo red positive and exhibited partial resistance to PK. Fish orally challenged with scrapie brain homogenate, on the other hand, developed only few, small, PrP-immunopositive brain aggregates which, however, were PAS and Congo red negative and PK sensitive. Collectively, these findings suggest that the novel proteinaceous deposition in the brains of prion-inoculated sea bream may serve as a starting point for further studies on fish susceptibility to TSEs.

A putative risk factor supporting prion transmission to fish could have been TSE-contaminated meat and bone meals (MBM) that were possibly used in aquaculture for years before the application of a total feed ban on the use of rendered mammalian proteins in feeds for farmed animals [17]. Thus, addressing the possibility of prion transmission to fish is an important task with great relevance to public health.

In this study we aimed at the evaluation of the possible conversion of piscine PrPs in a prion-infected cellular environment. Mouse neuroblastoma cells chronically infected with the mouse-adapted scrapie strain 22L were transiently transfected with three mature fish prion proteins, namely *Danio rerio* PrP-1 (ZebPrP-1), *Danio rerio* PrP-2 (ZebPrP-2) and *Sparus aurata* PrP-1 (SaurPrP-1) in the form of murine-piscine chimeric constructs. The intracellular distribution and a possible structural conversion of these proteins following interaction with the endogenous murine PrP^Sc^ were examined.

## 2. Results

### 2.1. Expression and Post-Translational Processing of Piscine Prion Proteins in Neuroblastoma Cells

**Figure 2:**
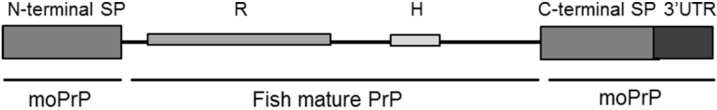
Chimeric mouse-fish constructs. Schematic illustration of the chimeric recombinant protein constructs introduced into the neuroblastoma cells. SP, signal peptide; R, repeat region; H, hydrophobic domain; 3'UTR, 3' untranslated region; moPrP, mouse PrP.

In this study, we used a well-established mouse neuronal cell line, namely neuroblastoma-2A cells (N2a) [19,20,21], permanently infected with the mouse-adapted scrapie strain 22L [22], in order to express and study three different piscine prion proteins. The coding sequences corresponding to the mature protein fragments of ZebPrP-1, ZebPrP-2 and SaurPrP-1 were introduced into pcDNA3.1 constructs flanked by the sequences coding for the murine PrP *N*- and *C*-terminal signal peptides (SP) (Figure 2). These constructs were used for the transfection of the N2a cells (both scrapie infected and non-infected). In order to estimate the transfection efficiency, a pcDNA3.1/Zeo(+) vector harboring the EGFP reporter was used, thereby calculating an uptake and expression percentage of approximately 70% (not shown).

Under physiological intracellular conditions, the *N*- and *C*- terminal signal peptides partly lead the biosynthetic and post-translational processing of PrP inside the endoplasmic reticulum (ER) and the Golgi apparatus. These events involve cleavage of the amino-terminal SP, addition of glycan chains at certain asparagine residues and attachment of a GPI-anchor following cleavage of the carboxy-terminal SP [23]. Thus, the integration of the piscine PrP coding sequences into the aforementioned mammalian nucleic acid cassettes aimed at ensuring mammalian-like biosynthesis, trafficking and topology of the teleost prion proteins in the murine neuronal cells.

**Figure 3:**
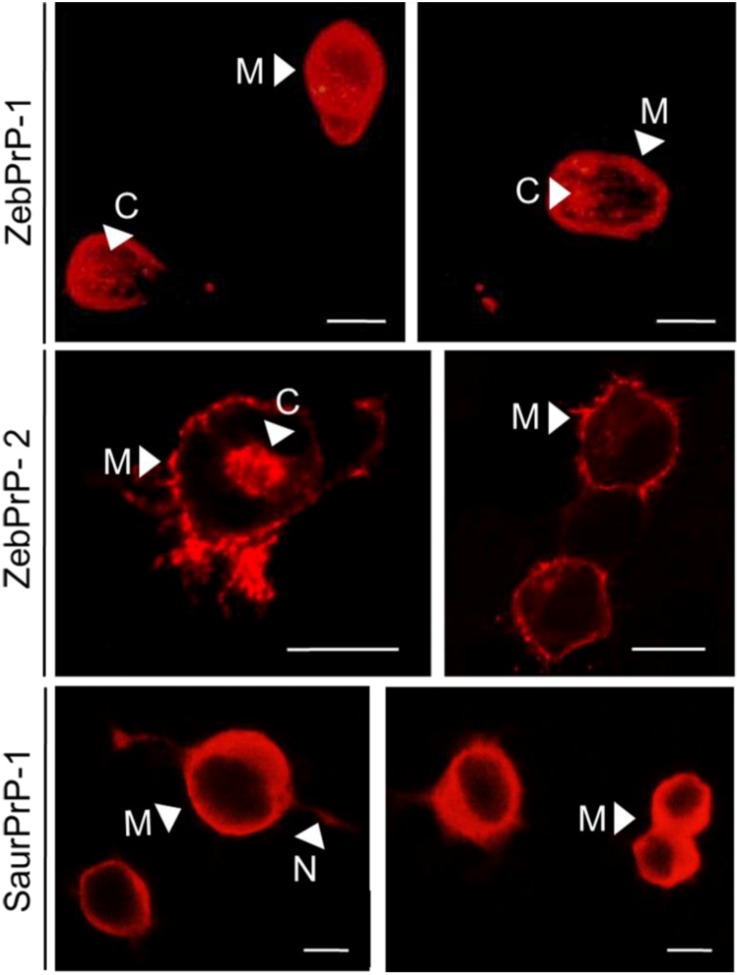
Indirect immunofluorescence of ZebPrP1, ZebPrP2 and SaurPrP1 in N2a cells indicates murine-like intracellular processing and localization. Fluorescent immunolabelling of N2a cells transfected with ZebPrP-1 and incubated with ZebPrP1 antiserum (1:750, upper panel), transfected with ZebPrP-2 and probed with ZebPrP2 polyclonal antibody (1:1500, middle panel) and, finally, transfected with SaurPrP-1 and incubated with SaurPrP1 antiserum (1:1000, lower panel). M, plasma membrane; C, cytoplasm; N, neurite. Scale bars, 10 μm.

To evaluate the cellular localization of the piscine prion proteins in non-infected N2a cells, we employed indirect immunofluorescence. Transfected cells were grown onto coverslips at 70%–80% confluence, probed with the appropriate anti-fish PrP antibody and finally incubated with the secondary fluorescent antibody (see Methods and Figure 3). Non-transfected N2a cells (both scrapie-infected and non-infected), or PrP-transfected cells that were not incubated with a specific anti-fish PrP antibody were used as negative controls, showing no positive signal (not shown). Intense immunoreactivity of all three recombinant piscine PrPs was detected at the plasma membrane of both single and adjacent cells, with the cell-cell contacts displaying particularly strong immunolabeling. Additionally, diffuse PrP-immunopositivity was observed intracellularly, in Golgi/trans-Golgi network, in cells transfected with each one of the three piscine proteins. PrP immunofluorescent labeling was similar among scrapie-infected and non-infected N2a cells, with the synaptically connected neurites, exhibiting notable PrP-immunopositivity in 22L-ScN2a cells (not shown). Our findings suggest that the fish prion proteins very likely underwent a mammalian-like biosynthetic process in the 22L-ScN2a cells, which resulted in their immunodetection both at the plasma membrane and intracellularly, where they might have been exposed to the endogenous PrP^Sc^.

**Figure 4:**
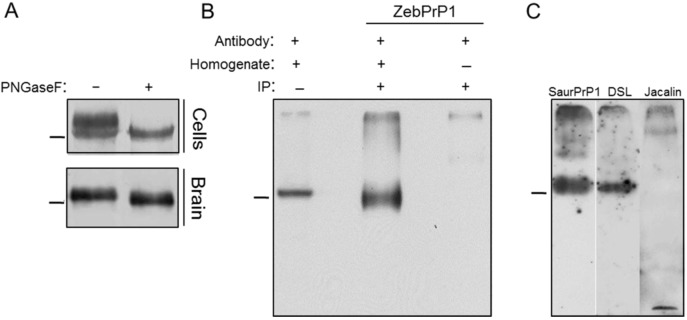
SaurPrP-1 is glycosylated *in vitro* and *in vivo*. (**A**) SaurPrP-1 transfected 22L-ScN2a cells (upper panel) or sea bream brain homogenate (lower panel) were treated (+) or not treated (−) with PNGaseF and then analyzed by Western blotting using SaurPrP1 antiserum (1:1000 and 1:20,000, respectively); (**B**) Isolation of SaurPrP-1 from sea bream brain by immunoprecipitation using ZebPrP1 antiserum (middle lane). Brain homogenate without immunoprecipitation treatment (left lane) and immunoprecipitation omitting the brain homogenate (right lane) were used as positive and negative controls, respectively; (**C**) Lectin staining of immunoprecipitated SaurPrP-1. Blotting with SaurPrP1 polyclonal antibody was used as positive control (left lane); the same immunoprecipitation fraction was probed with DSL (middle lane) and jacalin (right lane). Western blotting was performed using SaurPrP1 antiserum (1:20,000). Relative molecular mass markers, 47.5 kDa.

To further characterize the post-translational processing of SaurPrP-1 *in vitro*, we aimed at evaluating the glycosylation of the recombinant protein in the mammalian cells. Comparison of the electrophoretic mobility of SaurPrP-1 before and after digestion with PNGase F, an endoglycosidase that hydrolyzes *N*-glycan chains in glycoproteins, revealed that SaurPrP-1 gets similarly glycosylated in the 22L-infected (Figure 4A, upper panel) and non-infected N2a cells (not shown). In particular, the protein was found to be present in both a glycosylated and a non-glycosylated state, resulting in a two-band pattern on a Western blot. Following treatment with PNGaseF, the higher molecular weight band was abolished, indicating the removal of the carbohydrate group(s). A similar molecular weight shift was observed following the deglycosylation of native SaurPrP-1 in brain homogenate (Figure 4A, lower panel), demonstrating its glycoproteinaceous nature *in vivo*, as predicted previously [10]. Interestingly, endogenous SaurPrP-1 was found only in the higher-molecular-weight glycosylated state. Moreover, brain-derived immunoprecipitated SaurPrP-1 (Figure 4B) was positively stained with Datura stramonium lectin (DSL) (Figure 4C), which recognizes β-1,4 linked *N*-acetylglucosamine oligomers and is commonly used to discriminate between different mammalian PrP glycotypes [24]. In contrast, no staining was observed after incubation with jacalin (Figure 4C), a lectin that shows a high binding preference for *O*-linked glycans [25].

### 2.2. Evaluation of Piscine PrP Convertibility

**Figure 5:**
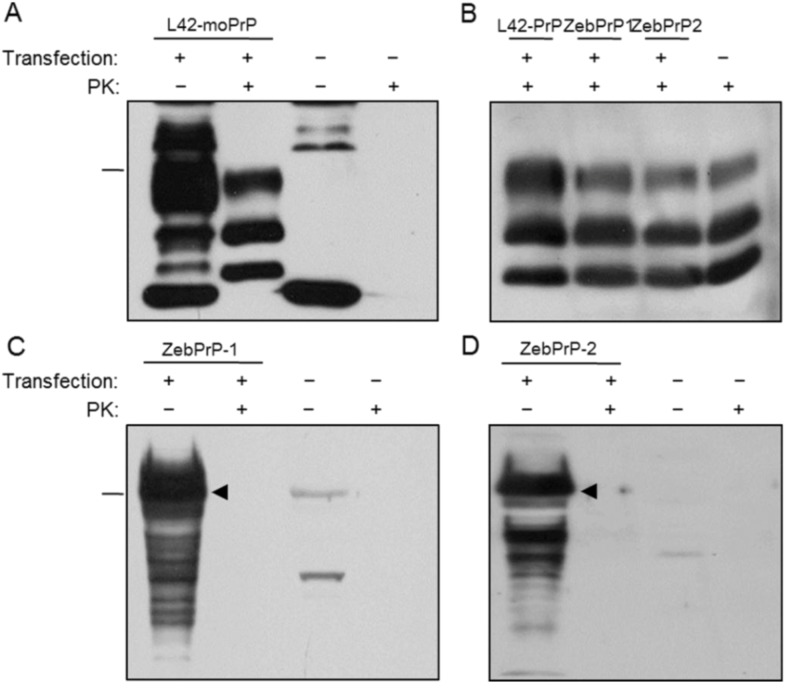
Mature ZebPrP1 and ZebPrP2 exhibit no PK-resistance in the 22L-ScN2a cell line. Western blot analysis of 22L-ScN2a cells transfected with mouse or fish recombinant proteins (transfection +) and non-transfected (transfection −) control cells. (**A**) Positive conversion control, cells transfected with L42-moPrP (L42, 1:10); (**B**) Endogenous PrP^Sc^, cells transfected with fish and mouse PrPs (6H4, 1:5000); (**C**) ZebPrP-1 conversion potential (ZebPrP1, 1:750); (**D**) ZebPrP-2 conversion potential (ZebPrP2, 1:1500). PK concentration, 5 μg/mL. Arrowheads indicate the fish proteins of interest. Relative molecular mass markers, 32.5 kDa (**A**,**B**), 62 kDa (**C**,**D**).

**Figure 6:**
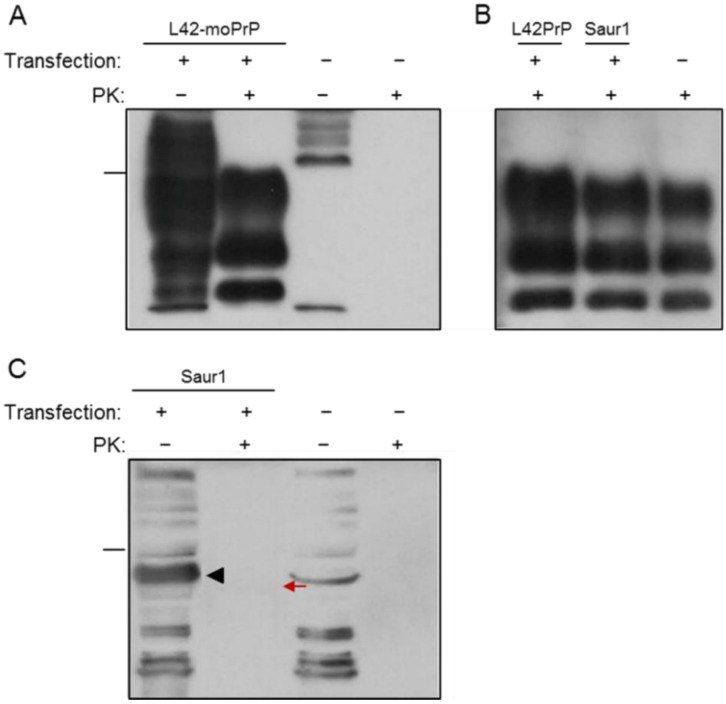
Evaluation of mature SaurPrP1 conversion potential in the 22L-ScN2a cell line. Western blot analysis of 22L-ScN2a cells transfected with mouse or fish recombinant proteins (transfection +) and non-transfected (transfection −) control cells. (**A**) Positive conversion control, cells transfected with L42-moPrP (L42, 1:10); (**B**) Endogenous PrP^Sc^, cells transfected with fish and mouse PrPs (6H4, 1:5000); (**C**) SaurPrP-1 conversion potential (SaurPrP1, 1:1000). PK concentration, 5 μg/mL. Arrowhead indicates recombinant SaurPrP-1. Red arrow indicates faint PK-resistant band. Relative molecular mass markers, 32.5 kDa (**A**,**B**), 62 kDa (**C**).

To examine the possibility of fish prion proteins getting converted by mammalian PrP^Sc^ into a structural, putatively pathogenic isoform, we assayed for their resistance to proteolytic digestion by PK, following exposure to the scrapie-infected cellular environment. To test the endogenous potential of our experimental system for homologous conversion, we used a pcDNA3.1 construct harboring L42-moPrP, a mutant murine prion protein carrying tyrosine instead of tryptophan at residue 144, a substitution that renders it differentially antigenic against the monoclonal antibody L42, which cannot recognize the wild type mouse PrP [26]. 22L-ScN2a cells transfected with L42-moPrP were able to efficiently convert the exogenously introduced homologous prion protein into a conformational isoform displaying PK-resistance, as indicated by the three-band pattern observed after treatment of the cell lysates with PK (Figure 5A and Figure 6A).

Cells transfected with each of the three piscine PrPs were initially analyzed to evaluate the endogenous PrP^Sc^ levels, theoretically reflecting their conversion potential. The results indicated that the expression of the three recombinant fish prion proteins did not appear to interfere and thereby affect the endogenous conversion mechanism of the cells (Figure 5B and Figure 6B). However, no evidence of resistance to proteolytic digestion of the studied fish PrPs was detected under conventional conditions of proteolysis and blot processing (Figure 5C,D and Figure 6C), as indicated by the absence of PK-resistant protein bands following Western blot analysis with the specific anti-fish PrP antisera. A barely visible band was detectable after PK digestion of Saur1 transfected cells (red arrow, Figure 6C). To explore the possibility of a partial resistance of SaurPrP-1 to PK digestion that would go undetected at a single—possibly too high—PK concentration, we treated lysates of transfected 22L-ScN2a cells with the proteolytic enzyme at lower, gradually increasing concentrations ranging from 0.1 to 1 μg/mL. We observed a specific to SaurPrP-1 immunoreactive band pattern at a PK concentration of 0.5 μg/mL (Figure S1). To further examine whether the difference observed could be attributed to a possible structural conversion of SaurPrP-1 by the endogenous cellular scrapie agent or it just reflected the expected gradual processing of the protein at such a low PK concentration, we performed the same treatment in both scrapie-infected and non-infected transfected cells. Following Western blot analysis, no difference was observed between scrapie-infected and non-infected N2a cells under these particular digestion conditions (Figure S1), suggesting the absence of a conformational, partially PK-resistant isoform of SaurPrP-1.

### 2.3. Sequence Analysis, Structural Predictions and PK Cleavage Site Simulation

To gain further insight into the PK-related behavior of fish PrPs and assess the possibility of variant PK proteolytic target distribution across vertebrate taxa and the *N*- and *C*-terminal domains of PrPs, we have examined general properties that might influence protein surface accessibility in a comparative sequence analysis context as a proxy for conformational diversity. Thus, the analysis was performed for prion proteins across vertebrate taxa in order to assess their relative propensities for PK-resistance.

**Figure 7:**
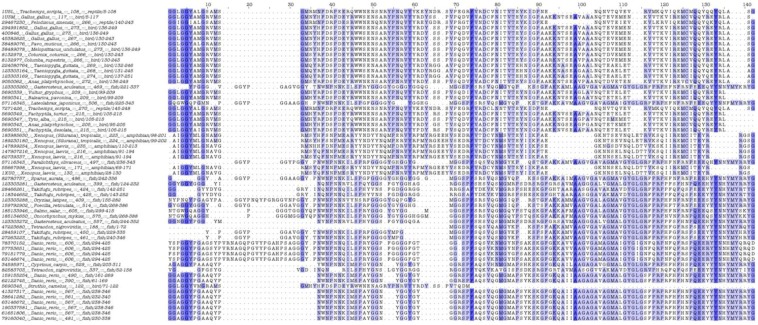
Alignment of the *C*-terminal region of 58 detected non-mammalian PrP sequences. The *C*-terminal domain of PrPs is shown, with identifiers, species names, total length, vertebrate taxon and sequence coordinates for each sequence (**left panel**). The full alignment is provided as Supplement 2 in FASTA format. Boxes signify sequence identity (darker color corresponds to more conserved positions). Note that the three sequences obtained from the PDB database, namely 1U5L (*Trachemys scripta*, reptile), 1U3M (*Gallus gallus*, bird) and 1XU0 (*Xenopus laevis*, amphibian) correspond to proteins of known structure.

Since only the *C*-terminal region of PrP has been structurally determined, we employed comparative sequence analysis, in order to accurately identify the *N*- and *C*-terminal regions of the PrP superfamily. Two sets of sequences, the FI-29 set (29 fish PrP sequences) and the VE-29 set (29 non-fish, non-mammalian vertebrate PrP sequences) were extracted from a master alignment (855 positions in length) and further used as reference sets for comparison (Figure 7, see also Materials and Methods). A similarly sized non-redundant set of mammalian sequences was obtained and compared against the non-mammalian sequences, further using proteins of known structure, in order to accurately delimit the *N*- and *C*-terminal regions.

Potential PK target sites were predicted using the ExPASy peptide cutter. Due to uncertainties corresponding to over-prediction by pattern searches, the acquired results are considered indicative. Nevertheless, over-prediction was a systematic error, consistently applied across the generated reference datasets. Moreover, since only natural sequences were used in order to alleviate issues of mutants and synthetic constructs, it is encouraging that highly similar sequences yield similar predictions (not shown), signifying that pattern searches for PK sites across PrPs might reflect a genuine biological effect.

**Figure 8:**
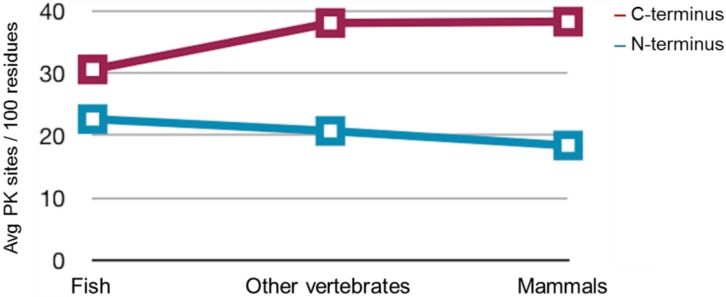
Comparison of normalized PK target sites in vertebrate taxa and PrP domains. Mean values of normalized PK target sites (average values of sites/100 residues—see Methods) across the three different vertebrate taxa that were examined, namely fish, other vertebrates (excluding fish or mammals) and mammals. The *N*-terminal region of PrPs is marked in blue, while the *C*-terminal region is marked in red. It is evident that the only statistically significant difference in these measurements arises from the lower value of the fish *C*-terminal region (see Text for details).

The predicted PK sites for each of the studied groups (fish, other vertebrates, mammals) corresponding either to the *N*-terminal or to the *C*-terminal regions of PrPs were averaged per hundred residues and plotted (Figure 8). The average values of the predicted PK sites per hundred residues in the *N*-terminal region of piscine, other vertebrate and mammalian PrPs were predicted to be 22.63, 20.72 and 18.39, respectively (Figure 8). In the *C*-terminal region, these values were found to be 30.60 per hundred residues for fish, in contrast to 38.06 and 38.27 for other vertebrates and mammals, respectively (Figure 8).

Nevertheless, piscine PrPs are significantly longer, thus, the actual total number of PK cleavage sites in both the *N*-terminal and *C*-terminal regions of piscine proteins is higher than the one predicted for mammalian PrPs. This is obvious when the total PK target sites predicted to be present in the *N*-terminal and *C*-terminal regions of mouse (Mo), *Sparus aurata* (Saur) and zebrafish (ZebPrP1, ZebPrP2) PrPs are considered (Table 1). In line with data presented in Figure 8, no significant differences in PK target sites per hundred residues are detected between murine and the three piscine PrPs under study at the *N*-terminal region, while more PK targets per hundred residues are predicted to be present in the *C*-terminal regions of murine PrP. However, when the total number of PK target sites is examined, it is evident that a higher number of PK sites is present in both the *N*- and *C*-terminal regions of piscine PrPs compared to murine PrP.

**Table 1:** Predicted PK target sites in the full-length sequences and along the *N*- and *C*-terminal regions of mouse (Mo), *Sparus aurata* (Saur), and zebrafish (ZebPrP1, ZebPrP2) PrPs.

Species	PrP length	PK sites	PK sites	PK sites	PK sites	PK sites
		(Total)	*N*-terminus (total)	*N*-terminus (/100 residues)	*C*-terminus (total)	*C*-terminus (/100 residues)
Mo	254	101	25	27.4	76	46.6
Saur	496	167	73	28.8	94	38.6
ZebPrP1	606	182	80	24.7	102	36
ZebPrP2	567	188	63	25.7	125	38.8

**Figure 9:**
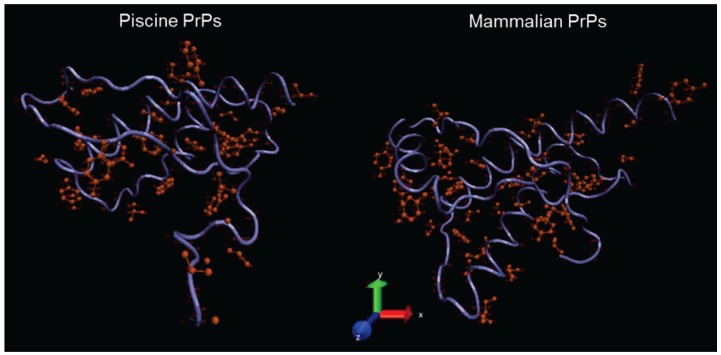
Fish (left) and mammalian (right) models of PrP *C*-terminal domain shown as ribbon trace, with putative PK sites highlighted with side chains (in red). Details about model construction and quality control are available in the Methods section.

We further looked for potential piscine and mammalian PrP differences regarding the accessibility of PK at its target sites. For this reason, structural prediction models corresponding to the *C*-terminal regions of the fish and mammalian consensus sequences were generated with concurrent visualization of the predicted PK cleavage sites (Figure 9).

**Figure 10:**
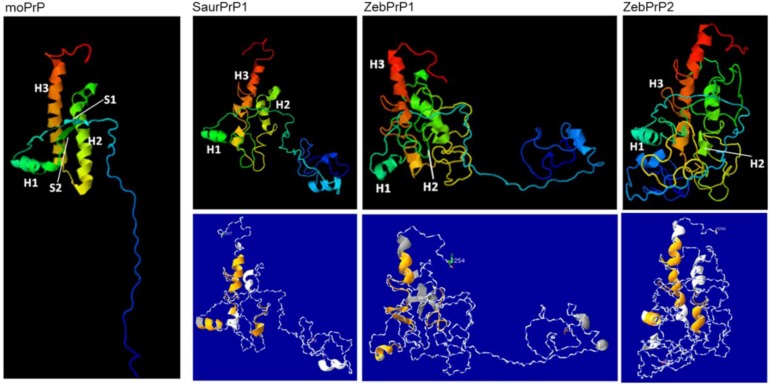
Structural prediction models corresponding to the *C*-terminal regions of the murine (moPrP) and the three piscine PrP proteins under study (SaurPrP, ZebPrP1, ZebPrP2). The well-defined secondary structure elements of murine PrP are shown (H1, H2, H3: helices 1, 2 and 3; S1, S2: sheets 1 and 2). Simplified views of the piscine models, highlighting (in orange) regions corresponding to murine PrP secondary structure elements are shown in blue background.

Finally, aiming at gaining some insight into the factors that might have affected the result of the interaction between the heterologous piscine mature PrPs and the endogenous murine PrP^Sc^ in the employed experimental system, individual structural prediction models corresponding to the *C*-terminal regions of the piscine prion proteins under study and to the murine PrP were generated (Figure 10, see also Materials and Methods). These models allowed assessment of similarities and/or differences in secondary structural modules that may be involved in the initial PrP^C^-PrP^SC^ interaction and/or in the actual conversion process. Among secondary structural elements, the region corresponding to the murine PrP helix 1(H1) was detected with the highest structural integrity in all three piscine PrPs under study.

## 3. Discussion

The occurrence of prion diseases has been reported in different mammalian species. In animals, prion diseases display an infectious nature, and result from the interaction of the endogenous host PrP^C^ with an exogenously introduced (mainly through the consumption of prion-infected food), aberrant PrP isoform, termed PrP^Sc^. To date, limited data are available regarding the possibility of other non-mammalian species, and in particular fish, to support a prion infection. Fish display two orthologues of mammalian PrP, and thus they theoretically may support the manifestation of prion diseases upon interaction with abnormal PrP^Sc^, possibly introduced through the consumption of BSE-contaminated meat and bone meals (MBM), that could have been used in aquaculture for years before the application of the total feed ban on the use of rendered mammalian proteins in feeds for farmed animals [17].

Since aquaculture represents a main sector for food production and fish constitute an easily interpretable experimental model, the study of the risk of prion transmission to fish is highly relevant not only to public health issues but also to fundamental principles of prion pathogenesis.

Cell culture models efficiently propagating a variety of rodent-adapted TSE strains are widely used to study and evaluate prion conversion, providing a mechanistic insight into the molecular events underlying prion transmissibility and pathogenesis [27]. In this work, we aimed at studying three different piscine PrPs within a prion environment. For this reason, we expressed chimeric proteins, consisting of the murine PrP *N*- and *C*-terminal signal peptides and the regions corresponding to the mature PrPs of ZebPrP-1, ZebPrP-2 and SauPrP-1, in a scrapie-infected mouse neuroblastoma cell line (22L-ScN2a). The presence of the murine PrP signal peptides in the fish chimeric proteins aimed at the appropriate biosynthesis, processing and trafficking of the exogenously expressed piscine proteins in the murine cell line. Immunoflueresence analysis showed that this approach resulted in a similar to mouse PrP [28] localization for all the piscine proteins under study.

With regard to cellular topology, early *in vitro* studies led to the conclusion that PrP^C^ is a membrane glycoprotein tethered to the lipid bilayer via its GPI anchor [20,29]. Since PrP^C^ recycles between the plasma membrane and the endocytic pathway, *in vitro* immunolocalization has involved several cellular compartments, including early and late endosomes, lysosomes, ER and Golgi [30]. The cellular topology of PrP^Sc^ is much more difficult to define, since the two PrP isoforms cannot be antigenically discriminated without chemically affecting cell morphology and, thus, interfering with the protein-organelle colocalization [27]. However, it has been shown that PrP^Sc^ is detected both at the plasma membrane and intracellularly, in endosomes, lysosomes [30], neurites [31], synapses [32], and even inside the nucleus, where it may interact with chromatin [33]. Overlapping localization between PrP^C^ and PrP^Sc^ within cellular compartments may serve as an indication of putative interaction and/or conversion sites, although the absolute topology of these events still remains to be elucidated [27].

Notably, a study demonstrated that localization of the zebrafish prion proteins at the plasma membrane of transfected N2a cells is responsible for their involvement in *trans*-interactions at cell-cell contacts, accounting for the triggering of a Src-related signaling pathway, which is highly active and thus, indispensable during embryogenesis and early neuronal development [13]. Interestingly, such interactions were observed even between cells expressing PrPs of heterologous origin (murine and piscine), indicating a possible inter-species association of prion molecules, that could under certain circumstances result in a pathogenic conversion event [34]. Finally, a study on subcellular localization and cell-cell adhesion of prion proteins demonstrated that zebrafish and mouse PrPs are both expressed in the same subcellular domains and cell-cell junctions in a pattern similar to what we observed upon overexpression of the zebrafish and sea bream mature PrPs in N2a cells [35]. Taken together, these data suggest that the recombinant piscine proteins may co-localize with the murine PrP^Sc^ in the 22L-ScN2a cells, thus making their interaction plausible.

PrP molecules undergo post-translational processing, which includes the addition of acetylglucosamine groups. Since the glycosylation of several piscine prion proteins, including the ones from sea bass, perch and zebrafish, has been demonstrated before [9,13,36], we further studied the SauPrP-1 recombinant protein, with regard to its post-translational processing in the used cell line, by determining the degree and type of its glycosylation. Analysis of cell lysates after transfection with the SauPrP-1 pcDNA3.1 vector, showed the presence of a glycosylated and a non-glycosylated form of the protein. Similar analysis performed on sea bream brain homogenate, showed the presence of a glycosylated band of SauPrP-1, suggesting that the mature protein is similarly processed *in vivo* and *in vitro*. Moreover, the use of different lectins revealed that piscine PrPs acquire *N*- instead of *O*-glycosylation, similar to the mammalian PrPs, indicating that they are efficiently imported into the ER [36]. These data agree with the *in silico* analysis of SaurPrP-1 sequence, predicting the existence of a single *N*-glycosylation—required “sequon” motif, namely the characteristic amino acid triad Asn-Arg-Thr, with asparagine at residue 389. Since both mammalian and piscine prion proteins contain *N*-linked acetylglucosamine groups, the partial glycosylation “homology” between piscine and mouse PrPs could serve as a potentially favorable factor contributing to interaction between the heterologous molecules [37]. Similarly to our findings, zebrafish PrP-1 has been shown to be imported into the ER lumen and to undergo *N*-glycosylation upon expression in N2a cells, exhibiting a high mannose and complex glycoform modification pattern [36]. Taken together, these data suggest that the expression of the piscine prion proteins in our system undergoes the physiological processing events, strongly resembling the pertinent processes involved in endogenous murine PrP expression.

In order to assess the effectiveness of the piscine PrP^C^ and the murine PrP^Sc^ interaction, we investigated whether the fish prion proteins acquire a different conformation, which could lead to a prion disease phenotype. As an indicator of such a conformational change we employed PK treatment of the piscine PrPs, to determine whether they can acquire partial resistance to this proteolytic enzyme, which is thought to be a hallmark of prion diseases. Since previous studies have shown that the introduction of a heterologous prion protein may block, via a dominant-negative effect, the conversion of the endogenous PrP^C^ in a prion-infected cell line [38], we examined the impact of the fish PrP expression on the endogenous murine PrP^Sc^ levels. In contrast to previous studies, our results showed that the piscine PrPs did not interfere with the conversion potential of the cells. However, no PrP^Sc^-related PK-resistant fish PrP bands were detected, suggesting that either no effective interaction between the piscine and murine PrP isoforms occurred or that the piscine PrPs underwent a structural conversion, yet without displaying PK-resistance.

To further investigate the absence of PK-resistance of the piscine PrPs, we assessed the possibility of variant PK target distribution in the *N*- and *C*-terminal domains of PrPs across vertebrate taxa. Analysis of the predicted PK target sites showed that piscine PrPs have in total more PK cleavage sites due to the actual size of the mature molecules. In addition, we assessed the possibility of differential enzyme accessibility to its target sites, by generating structural prediction models of piscine and mammalian PrP *C*-terminal regions, and by visualizing the predicted PK sites on these structures. This kind of analysis demonstrates that piscine prion proteins are predicted to possess less well-defined secondary structural elements compared to mammalian PrPs. The higher degree of disordered regions predicted for the piscine PrPs may result in higher accessibility of the proteolytic enzyme to its target sites.

Even though not well understood, efficient interspecies prion transmission requires at least two steps: (a) initial interaction between the heterologous PrP^C^ and PrP^Sc^ molecules and (b) subsequent successful conversion of the normal PrP^C^ to PrP^Sc^ [39]. Both primary sequence homology and structural similarities between the interacting PrP molecules have been shown to determine the outcome of such an interaction [40]. Various studies aimed at the determination of the sites involved in the initial binding of PrP^C^ and PrP^Sc^ molecules and the subsequent PrP^C^ conversion. These sites are found at both the *N*-terminal and *C*-terminal part of PrP, the latter including helix 1 (H1) [41,42,43]. Some of these regions were also found to be involved in the actual conversion process [43,44].

Based on these data, one would expect that successful binding and conversion of heterologous PrPs would possibly occur upon significant structural similarity in the aforementioned regions between the interacting molecules. Structural models of the *C*-terminal region of the piscine PrPs under study, predict a generally higher degree of disordered regions compared to the murine PrP. Interestingly, the highest structural integrity was detected in piscine PrP regions highly resembling the well-defined H1 of murine PrP.

The inability to detect any PK-resistant moieties originating from the murine-piscine chimeras expressed in our study system may reflect either ineffective binding or ineffective conversion of these proteins by the endogenous PrP^Sc^. Taken that a structure similar to murine H1 is predicted to be present in the proteins under study and that this region has been implicated to participate in the initial binding of PrP^C^ to PrP^Sc^, we would not rule out the possibility that an initial binding of the murine-piscine chimeras did occur, though in a less efficient manner compared to the binding of the homologous molecule. An altered, not previously described molecular association between the heterologous molecules cannot be excluded either.

Moreover, the strain effect should also be considered, as prion strains display different PrP^Sc^ structures. The conformational selection model described by Collinge and Clarke [45] postulates that PrP molecules of a given species may accommodate only a particular subset of prion strain conformations. If subsets of two species overlap, transmission may occur. Thus, it could be the case that in our experimental system the mouse-adapted 22L scrapie strain may not drive the conversion of the studied proteins, due to the higher divergence between the interacting molecules.

The structural conversion of PrP^C^ to PrP^Sc^ renders most of the putative PK sites, and especially the ones found at the *C*-terminal region of the protein, inaccessible by the enzyme. Thus, cleavage mainly occurs at the unstructured *N*-terminal region, allowing for the detection of the PK-resistant core. Piscine PrPs are predicted to have arithmetically more PK cleavage sites both at their *N*-terminal and at *C*-terminal regions than the corresponding murine PrP, due to their longer amino acid sequence. Even though the regions involved in a structural rearrangement of piscine PrPs that would lead to a PK-resistant core cannot be speculated, the originally predicted highly disordered structure of these molecules would be expected to be preserved at some degree, and thus allow easier enzyme accessibility to the whole molecule. In this case, the higher numbers of putative PK cleavage sites in the piscine PrPs could explain our experimental results. This would also suggest that the inability to detect PK-resistance cannot rule out at this stage the possibility of any conformational change imposed to the proteins under study by the cellular PrP^Sc^.

It is noteworthy that cases of abnormal prion accumulation without accompanying resistance to proteolytic digestion have been frequently reported. More specifically, PrP^Sc^ aggregates of lower molecular weights, lacking the characteristic PK-resistant phenotype, have been previously identified in scrapie-infected N2a cells, where they co-existed with larger PK-resistant aggregates [46]. Additionally, *in vivo* findings support the existence of previously unidentified clinical TSE cases with no detectable brain PrP^Sc^ [47]. Moreover, our previous work demonstrated the presence of few, small, PrP-immunopositive brain aggregates which, however, were PAS- and Congo red-negative and PK-sensitive, in the brains of sea bream, which were orally challenged with scrapie brain homogenate [18].

Our study shows that in this particular experimental approach, piscine chimeric proteins do not acquire PK-resistance upon contact with the scrapie-derived murine PrP^Sc^, while no claims can be currently made on their pathogenic conversion potential. However, the outcome of a possible interaction between other TSE strains and certain piscine prion proteins cannot be predicted in advance and further studies are required in order to widely assess the risk of fish susceptibility to prion diseases [17].

## 4. Materials and Methods

### 4.1. Generation of DNA Constructs

The genes coding for ZebPrP-1, ZebPrP-2 and SaurPrP-1 proteins were amplified by standard polymerase chain reactions. As template, genomic DNA was used in the case of ZebPrP-1 and ZebPrP-2, whereas plasmid DNA (generous offer by Dr. G. Krey, National Agricultural Research Foundation, Fisheries Research Institute, Nea Peramos, Greece) was used in the case of SaurPrP-1. The mature coding sequences of ZebPrP-1, ZebPrP-2 and SaurPrP-1 comprise the nucleic acids 70-1740, 54-1617 and 79-1425, respectively. The DNA3.1/Zeo(+) vector was used for both the control (L42moPrP/pcDNA3.1 and EGFP/pcDNA3.1) and the fish constructs. The generation of the mouse-fish DNA cassettes was performed by standard restriction digestion and subcloning procedures. The final ZebPrP-1, ZebPrP-2 and SaurPrP-1 constructs included the mature piscine protein sequence (aa 24-580, 18-539 and 26-475, respectively), flanked by an *N*-terminal mouse PrP signal peptide (moPrP_1-22_) and a *C*-terminal mouse PrP GPI-anchor signal peptide (moPrP_228–255_), followed by the mouse PrP 3'UTR. The primer sequences used for the cloning of fish coding sequences into the mouse cassettes were the following: 5' AAGAATTCTGGGAAAGAAAGGCACTG 3' and 5' AACTCGAGACTTCTGGGAATTTTC 3' for ZebPrP-1, 5' AAGAATTCTGGCCAAACGCGGTGGTG 3' and 5' AACTCGAGACTACCGTTTCTGGCTTC 3' for ZebPrP-2 and 5' AGCGAATTCTGAAAAAAGGTGGCAGC 3' and 5' AACCTCGAGGCTCAGTGGGCTG 3' for SaurPrP-1.

### 4.2. Antibodies and Lectins

The three anti-fish polyclonal antibodies were used as previously described [18]. 6H4 monoclonal antibody was purchased from Prionics (Schlieren, Switzerland), goat-anti-rabbit- and goat-anti-mouse-HRP conjugated antibodies from Pierce (Thermo Fisher Scientific, Waltham, MA, USA) and goat-anti-rabbit Alexa Fluor 568 from Invitrogen (Grand Island, NY, USA). L42 monoclonal antibody [26] was kindly provided by Dr. M. Groschup (Institute for Novel and Emerging Infectious Diseases, Federal Research Institute for Animal Health, Insel Riems, Germany). Biotinylated lectins were purchased by Vector Laboratories Inc. (Burlingame, CA, USA).

### 4.3. Cell Culture and Transfections

Cells were cultivated as previously described [48] and transfected by a non-liposomal method, using FuGENE 6 Transfection reagent (Roche, Mannheim, Germany), according to the manufacturer’s instructions.

### 4.4. Trypsinization, Lysis and Proteinsase K Treatment

For trypsin treatment, intact cells were rinsed once in PBS and then incubated with trypsin (0.25%, *w*/*v*) for 2 min at RT. Cells were split at a ratio of 1:30 and after 24 h they were transfected. Three days after transfection, the cells were lysed. For lysis, cells were rinsed once with PBS and then incubated with ice-cold lysis buffer (Tris 10 mM (pH 7.5), NaCl 100 mM, EDTA 10 mM, Triton-100 0.5%, Na-Deoxycholate 0.5%) for 10 min at RT. Cell lysates were centrifuged for 1 min at 14,000 rpm. Half of each supernatant was methanol-precipitated after the addition of 5 mM PMSF, while the rest was PK treated. For PK-treatment, cell lysates were incubated with 5 μg/mL PK for 1 h at 37 °C. The digestion was stopped by the addition of 5 mM PMSF. Finally, 1% lauryl-sarcosyl was added and the proteins were methanol precipitated as before. Following centrifugation at 4000 rpm for 40 min, the protein pellets were briefly washed in 25 mM Tris (pH 8.8) containing 0.05% lauryl-sarcosyl and then centrifuged at 4000 rpm for 10 min. All the pellets were solubilized in 2.5× O’Farrell loading buffer, heated for 10 min at 100 °C and then analyzed by SDS-PAGE and Western blotting.

### 4.5. Western Blot Analysis

Following electrophoresis, the proteins were electrotransferred onto a PVDF membrane. The membrane was blotted with blocking buffer (5% powder milk in PBST) for 1 h at RT, then incubated with the primary antibody at 4 °C for 16 h and finally with the appropriate secondary antibody for 1 h at RT. The blots were developed using the ECL Western blotting substrate (Pierce, Thermo Fisher Scientific, Waltham, MA, USA), according to the manufacturer’s instructions.

### 4.6. Lectin Staining

0.4 mg brain equivalents were analyzed by SDS-PAGE and then electrotransferred onto a PVDF membrane. Blocking was performed in BSA 0.3% for 1 h at RT and following washes with PBS, the proteins were probed with the appropriate lectin (1 μg/mL) for 1 h at RT. The membrane was washed with PBS and incubated with ABC complex for 30 min at RT. Following washes, the blot was developed using the ECL Western blotting substrate (Pierce, Thermo Fisher Scientific, Waltham, MA, USA), according to the manufacturer’s instructions.

### 4.7. Indirect Immunofluorescence

Cells were grown on glass coverslips to a 70%–80% confluence, rinsed twice with PBS and then fixed in 4% paraformaldehyde for 30 min at RT. Permeabilization and blocking were performed by incubation in blocking buffer (gelatin 0.2%, TritonX-100 0.3%) for 30 min at RT. Cells were then rinsed with PBS and incubated with the appropriate primary antibody in blocking buffer for 1 h at RT, washed with PBS and probed with the fluorescent secondary antibody for 1 h at RT. The washed coverslips were mounted on glass slides and examined by fluorescence microscopy (Nikon Eclipse TE2000-4, Nikon Digital Sight DS-SMc, Nikon Instruments Inc., Melville, NY, USA).

### 4.8. PNGase Treatment

0.85 mg brain equivalents were pretreated with the appropriate buffers and finally with 2500 units of the enzyme (New England Biolabs, Ipswich, MA, USA), according to the manufacturer’s instructions, for 16 h at 37 °C. Following incubation, the appropriate volume of O’Farrell loading buffer was added and the sample was analyzed by SDS-PAGE and Western blotting as above. A similar procedure was applied in cell lysates.

### 4.9. Immunoprecipitation

30 μL of the polyclonal antiserum were mixed with 50 μL of 50% alcohol sepharose bead solution, the volume was adjusted to 500 μL by the addition of PBS containing 0.5% NP-40 (*w*/*w*) and 0.5% DOC (*w*/*w*), and the mixture was incubated on a rotating platform for 16 h at 4 °C. Following a brief spin at 5000 rpm, and washes with PBS, the sample was incubated with 4 mM DSS (disuccinimidyl suberate) ligand for 1 h at 4 °C. Blocking was performed in 50 mM Tris.HCl (pH 7.6) for 1 h at 4 °C and then the beads were washed with 0.1 M Glycine (pH 2.3), Tris.HCl (pH 7.6). Incubation with the brain homogenate (6 mg brain equivalents) was carried out for 16 h at 4 °C. Following washes with PBS, the beads were boiled in O’Farrell loading buffer for 10 min and briefly centrifuged. β-mercaptoethanol (5%) was added to the supernatant and finally the immunoprecipitated fraction was analyzed by SDS-PAGE and Western blotting as above.

### 4.10. Sequence Analysis and Comparison

Sequence searches against the non-redundant protein sequence database (NRDB) at the NCBI [49] were performed using PSI-BLAST [50] with default parameters and the *Sparus aurata* PrP as query (GI: 82780737). Compositionally biased regions were masked using CAST with a threshold value of 20 and otherwise default parameters [51], where relevant. After PSI-BLAST convergence, the query identifies 29 homologous sequences, all from fish species (called the FI-29 set). In this mode, no homologues of known structure can be retrieved until convergence, even with modified parameters for increased sensitivity due to sequence variability as well as the relatively confined taxonomic specificity. To enrich the reference sequence datasets with proteins of known structure, database searches as above were performed with the turtle prion sequence (PDB code: 15UL), a structure of “lower” taxonomic rank from a reptile species. Following 7 iterations, PSI-BLAST detects all known non-mammalian sequences, 65 in number. After removing seven synthetic constructs, the remaining 58 sequences are partitioned into two groups, 29 from fish (corresponding to the FI-29 set) and 29 from the other three non-mammalian vertebrate classes (called the VE-29 set), namely amphibians (7 sequences), reptiles (3 sequences) and birds (19 sequences)—Supplement 2. A similar analysis with more than 500 mammalian sequences was performed, using the *Sparus* *C*-terminus (residues 251–459) as a query against mammals only; after eliminating redundancy at 95% sequence identity, 54 mammalian sequences were obtained—Supplement 3. All sequence alignments were performed by CLUSTAL [52] and visualized by JalView [53]. Mammalian sequence redundancy was detected and eliminated by CD-HIT [54].

### 4.11. PK Cleavage Site Simulation

PK cleavage site simulation was performed by PeptideCutter on the ExPASy website for PK sites only [55] after degapping all sequences [56]. Cleavage simulation was obtained as the mean number of PK sites per 100 residues and further averaged over all sequences under consideration, for the three taxonomic divisions and the *N*- and *C*-terminal regions, resulting into 6 indicative numbers (see Figure 8). These values were subjected to Analysis of Variance (ANOVA) to test the null hypothesis that differences may arise by chance. A similar analysis was also narrowed down to the three piscine PrPs under study, namely *Sparus aurata* PrP1 (SaurPrP-1, UniProtKB/Swiss-Prot: Q2PZB0), zebrafish PrP1 (ZebPrP-1, UniProtKB/Swiss-Prot: Q3BDW0), zebrafish PrP2 (ZebPrP-2, UniProtKB/Swiss-Prot: Q50J88) and to the murine PrP (moPrP, UniProtKB/Swiss-Prot: P04925), in order to determine the actual number of putative PK cleavage sites of the full length molecules as well as their distribution in their *N*-terminal and *C*-terminal regions (see Table 1). Piscine PrP *C*-terminal regions (residues 254-459, 246-531, and 324-577 for SaurPrP-1, ZebPrP-2 and ZebPrP-1, respectively) were determined according to the corresponding murine PrP region, based on primary sequence alignment and secondary structural motifs.

### 4.12. Structure Prediction and Modeling

Alignment-based structure prediction was performed by Swiss-Model, with default parameters [57]. Models were generated as follows: consensus sequences from master alignments (fish, mammals) were obtained and screened against the NRDB databases using PSI-BLAST as above, to establish that corresponding regions properly identify their structural homologues (or not, in the case of the *N*-terminal domain). Both consensus sequences were subjected separately to Swiss-Model, the mammalian consensus against the human structure (105 residues long) and the non-mammalian consensus against the turtle structure (87 residues long). Structure superposition was performed by MultiProt [58], DALI [59] and PROMALS3D [60] and visualization by Jmol [61]. PK sites were visualized following prediction by PeptideCutter (see Figure 9). The protein models, structural superimpositions, PK mapping sites and additional data are available on request. A similar structural prediction analysis, narrowed down to the *C*-terminal regions of the piscine PrPs studied here (SaurPrP-1, UniProtKB/Swiss-Prot: Q2PZB0, residues 254-459, zebrafish PrP1 (ZebPrP-1, UniProtKB/Swiss-Prot: Q3BDW0, residues 324-577, and zebrafish PrP2, ZebPrP-2, UniProtKB/Swiss-Prot: Q50J88, residues 246-531) and the murine PrP *C*-terminal region (moPrP, UniProtKB/Swiss-Prot: P04925, residues 92-236), was also applied (see Figure 10). The analysis was performed using the Phyre2 software [62]. For the latter models no PK site visualization was performed.

## Supplementary Files

Supplementary File 1

Supplementary File 2

Supplementary File 3
