# Supplementary material for: Assessing Proteinase K Resistance of Fish Prion Proteins in a Scrapie-Infected Mouse Neuroblastoma Cell Line
Source: Viruses. 2014 Nov 13;6(11):4398–421. doi: 10.3390/v6114398 (PMC4246229; doi:10.3390/v6114398)
Supplement: Supplementary File 1 [file viruses-06-04398-s001.pdf]

Supplementary Material

Assessing Proteinase K Resistance of Fish Prion Proteins in a Scrapie-Infected Mouse Neuroblastoma Cell Line

Evgenia Salta, Eirini Kanata, Christos A. Ouzounis, Sabine Gilch, Hermann Schätzl and Theodoros Sklaviadis

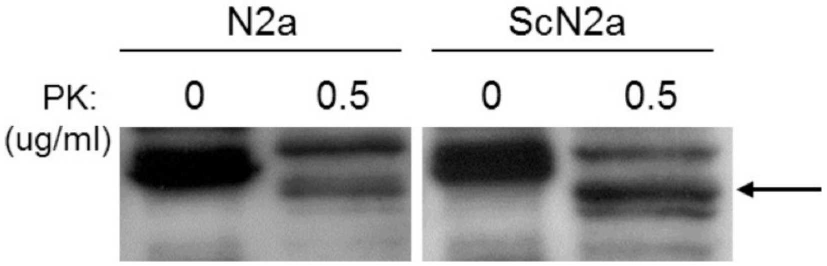

Figure S1. The SauPrP-1 immunoreactive band detected after digestion with lower PK concentration does not correspond to a PrP^{Sc}-related PK-resistant moiety. Western blot analysis of non-PK treated (0) and PK-treated (0.5) lysates from non-infected (N2a) and infected (ScN2a) cells expressing the SaurPrP-1 recombinant protein (SaurPrP1, 1:1000). PK concentration, 0.5 µg/mL. The arrow indicates the band discussed in the text.
